# Measuring, mapping and making sense of Irish health system performance in the recession

**DOI:** 10.1186/1472-6963-14-S2-P12

**Published:** 2014-07-07

**Authors:** Sara Burke, Stephen Thomas, Sarah Barry

**Affiliations:** 1 Centre for Health Policy and Management, Trinity College Dublin, Dublin, Ireland

## 

A new Irish government came to power in March 2011 during one of the most severe economic crisis experienced by any OECD country since the 1930s. This government promised the most radical proposals for health system reform in the history of the Irish state, including universal access to healthcare based on need not income, free GP care for all by 2015 and the introduction of Universal Health Insurance after 2016. All these reforms were to be achieved amidst the most severe cuts to the health budget. While the public health budget quadrupled between 1997 and 2007, since 2007 the Health Service Executive (HSE) – the public health system has been cut by 17.5% and there are 12,000 fewer staff in the health system.

The authors collected indicators of performance of the Irish health system during the economic crisis from 2005 to 2014, showing pre and post crisis trends. The authors assess how well the system has coped with a downsizing of resources by an analysis of a range of performance indicators:

(i) healthcare funding and resources,

(ii) the coverage of the population with subsidised care,

(iii) the efficiency of resource use,

(iv) access to timely care.

These indicators are used at an OECD level to assess health system performance. Ireland has a poor track record in data collection, especially in the health system, and that which exists comes from a wide range of sources including the Department of Finance Revised Estimates, HSE Annual Reports, HSE National Service Plans and waiting list data collected by the HSE. The data have been gathered in one place for the first time.

These show a system that managed ‘to do more with less’ from 2008 to 2012. They also demonstrate a system that was ‘doing more with less’ by transferring the cost of care onto people and by significant resource cuts. From 2013, the indicators show a system that has no choice but ‘to do less with less’. They indicate diminishing returns from crude cuts, evident in declining hospital cases, increased wait-times, as well as cuts to home care hours and increasing costs of agency staffing. The results suggest a limited window of benefit from austerity beyond which cuts and rationing prevail without structural change.

